# Prevalence and incidence of emergency department presentations and hospital separations with injecting-related infections in a longitudinal cohort of people who inject drugs

**DOI:** 10.1017/S0950268823001784

**Published:** 2023-11-13

**Authors:** Stephanie J. Curtis, Samantha Colledge-Frisby, Andrew J. Stewardson, Joseph S. Doyle, Peter Higgs, Lisa Maher, Matthew Hickman, Mark A. Stoové, Paul M. Dietze

**Affiliations:** 1 Disease Elimination Program, Burnet Institute, Melbourne, VIC, Australia; 2 Department of Infectious Diseases, the Alfred Hospital and Central Clinical School, Monash University, Melbourne, VIC, Australia; 3 National Drug Research Institute, Curtin University, Melbourne, VIC, Australia; 4 Department of Public Health, La Trobe University, Bundoora, VIC, Australia; 5 The Kirby Institute, Faculty of Medicine, UNSW, Wallace Wurth Building, Kensington, NSW, Australia; 6 Population Health Sciences, Bristol Medical School, University of Bristol, Bristol, UK; 7 School of Public Health and Preventive Medicine, Monash University, Melbourne, VIC, Australia

**Keywords:** bloodstream infection, endocarditis, hospitalization, skin and soft tissue infections, substance-related disorders

## Abstract

People who inject drugs are at risk of acute bacterial and fungal injecting-related infections. There is evidence that incidence of hospitalizations for injecting-related infections are increasing in several countries, but little is known at an individual level. We aimed to examine injecting-related infections in a linked longitudinal cohort of people who inject drugs in Melbourne, Australia. A retrospective descriptive analysis was conducted to estimate the prevalence and incidence of injecting-related infections using administrative emergency department and hospital separation datasets linked to the SuperMIX cohort, from 2008 to 2018. Over the study period, 33% (95%CI: 31–36%) of participants presented to emergency department with any injecting-related infections and 27% (95%CI: 25–30%) were admitted to hospital. Of 1,044 emergency department presentations and 740 hospital separations, skin and soft tissue infections were most common, 88% and 76%, respectively. From 2008 to 2018, there was a substantial increase in emergency department presentations and hospital separations with any injecting-related infections, 48 to 135 per 1,000 person-years, and 18 to 102 per 1,000 person-years, respectively. The results emphasize that injecting-related infections are increasing, and that new models of care are needed to help prevent and facilitate early detection of superficial infection to avoid potentially life-threatening severe infections.

## Background

Injecting-related infections (IRIs) are a clinical complication of injection drug use. IRIs among people who inject drugs are most commonly localized skin and soft tissue infections (SSTI), or can be more invasive systemic infections, such as bacteraemia, osteomyelitis, and endocarditis [1]. SSTI can often be managed in primary care; however, if initial treatment is delayed, acute treatment in the emergency department (ED) may be required [2, 3]. Invasive infections usually require hospital care and can result in lengthy hospital stays for complex surgical intervention and prolonged intravenous antibiotic therapy [1, 4]. IRIs account for a high burden of disease and a substantial proportion of the total hospitalizations of people who inject drugs, and there is evidence that hospitalizations for all types of IRI are increasing globally [5–13].

There are few estimates of the incidence of IRIs among people who inject drugs, with empirical evidence of IRIs in this group restricted largely to prevalence estimates from people engaged in drug treatment or self-report data [11, 12]. A systematic review estimated a varied lifetime prevalence of IRI in people who inject drugs; SSTI (6–69%), sepsis/septicaemia (2–10%), infective endocarditis (0.5–12%), and bone and joint infections (0.5–2%) [2]. In a recent survey of people who inject drugs in Victoria, Australia, 24% self-reported having an injection-related health issue in the preceding month, including 7% reporting infection and/or abscess [14]. However, as indicated, most estimates are derived from self-reported data and validity of estimates may be questioned in the absence of clinical confirmation of these infections [2].

Hospitalization data, typically coded using International Classification of Diseases (ICD) codes, can be used to ascertain the incidence of IRIs requiring clinical care that are likely related to injecting drug use [12]. In Australia, incidence estimates of hospital separations with an IRI are limited to a cohort of people with a history of opioid agonist treatment in New South Wales (43 per 1,000 person-years between 2001 to 2017) and a cohort identified by an algorithm of ICD, Tenth Revision, Australian Modification (ICD-10-AM) codes at a Melbourne health service (192 per 100,000 annual hospital admissions between 2008 and 2018) [12, 13]. International incidence estimates of hospital separations with an IRI are also limited to a cohort of people who injected heroin entering community-based substance use treatment in South London (73 per 1,000 person-years between 2006 and 2017) [11] and people who inject drugs with problematic alcohol use in a Swedish Prison and Probation Service (SSTI, 28.3 per 1,000 person-years and systemic bacterial infection, 9.1 per 1,000 person-years between 2001 and 2014) [15]. People who inject drugs with a history of opioid agonist treatment are not necessarily representative of the broader community of people who inject drugs; approximately half of people who inject drugs have engaged in opioid agonist treatment in their lifetime in Australia [16]. Additionally, cohorts of people who inject drugs engaged in opioid agonist treatment are likely to underestimate the incidence of hospitalizations as they are likely to inject less frequently compared to those not engaged in opioid agonist treatment and are likely to be more engaged with healthcare services which may be protective of infection [17].

Overall, there is limited evidence on IRI incidence among people who inject drugs and despite evidence that IRIs are an increasing issue for people who inject drugs, few have investigated incidence of infections among a community cohort of people who inject drugs. In this study, we address this gap by examining the incidence and period prevalence of ED presentations and hospital separations related to IRI in a cohort of people who inject drugs in Melbourne, Australia.

## Methods

### Study design

We performed a descriptive analysis of a prospective observational cohort linked to administrative health data from 2008 to 2018. The full details of the cohort study, the Melbourne Injecting Drug User Cohort Study (SuperMIX), can be found elsewhere [18, 19].

### Setting and participants

A total of 1,288 SuperMIX participants were included who resided in Melbourne metropolitan or the Greater Geelong region, Victoria, Australia. Participants were aged 18 or over, held a valid Australian universal healthcare insurance (Medicare) number, and injected either heroin or methamphetamine at least monthly over the six months prior to baseline recruitment. Participants were recruited through a combination of respondent-driven sampling, snowball sampling, and street-based outreach methods in multiple recruitment waves between January 2008 and July 2019. Primary data collection included socio-demographics (e.g., age, sex, country of birth, Aboriginal and Torres Strait Islander status, employment status, education, housing, and history of incarceration), drug behaviours (e.g., age at first injection, type, and frequency of drug use), and health-related characteristics (e.g., health service utilization, interactions with criminal justice systems and validated well-being assessment tools). Participants completed optional annual follow-up surveys following recruitment to further capture socio-demographics, drug behaviours, and health-related characteristics [20, 21].

### Data sources

Primary cohort data were linked to state-wide hospital data from the Victorian Emergency Minimum Dataset and Victorian Admitted Episode Dataset, and data on all deaths in Australia from the National Death Index. The Victorian Emergency Minimum Dataset details administrative and clinical data on ED presentations to Victorian public hospitals. The Victorian Admitted Episode Dataset details administrative and clinical data on hospital separations at Victorian public and private hospitals, including ‘hospital in the home’ (admitted acute care provided to patients in their own home, or other suitable environment outside hospital). ICD-10-AM codes are used to code up to three diagnoses per ED presentation in the Victorian Emergency Minimum Dataset and up to 39 diagnoses per hospital separation in the Victorian Admitted Episode Dataset. The National Death Index collates information on cause (ICD-10-AM) and date of death in Australia and was used to determine all-cause mortality following ED presentation or hospital admission and to censor participants when calculating person-years. Data were linked through deterministic linkage as described elsewhere [20], and all participants had consented to retrospective data linkage before their enrolment date to allow for linkage from 1 January 2008 onwards, regardless of the participants’ age at the time of ED presentation or hospital separation. Although this is a prospective observational cohort, data linkage from 2019 onwards was not yet available at the time of analysis.

### Outcome variables

ED presentations and hospital separations with an IRI were the outcomes, identified using ICD-10-AM codes in the Victorian Emergency Minimum Dataset and Victorian Admitted Episode Dataset. The ICD-10-AM codes were selected based on a previous study of IRIs in the same setting (Supplementary Table S1) and were identified as ED presentation or hospital separations with any IRI coded (i.e., either as a primary or secondary diagnostic code) [13]. In Australia, dedicated hospital staff are trained to retrospectively review clinical notes and encode ICD-10-AM data according to strict criteria for the Victorian Admitted Episode Dataset. For the Victorian Emergency Minimum Dataset, computer software is used to encode ICD-10-AM data by mapping the clinical diagnoses entered by clinicians in patient discharge documentation [21]. IRIs identified through the ICD-10-AM codes were grouped as SSTI and invasive infections (bloodstream infection/sepsis, osteomyelitis/septic arthritis, and infective endocarditis). Complicated SSTIs were defined as patients that had an invasive infection alongside a SSTI, and uncomplicated SSTIs were defined as patients with an SSTI but no invasive infection.

### Additional cohort characteristics

A range of additional data were collected from the data sources. Primary cohort data collected date of birth, sex, country of birth, and Aboriginal and Torres Strait Islander status. Where primary cohort data were missing date of birth or sex, we utilized the Victorian Emergency Minimum Dataset and Victorian Admitted Episode Dataset data. The Victorian Emergency Minimum Dataset and Victorian Admitted Episode Dataset collected date of most recent prior ED presentation and hospital separation, hospital admission or discharge following ED presentation, hospital admission source (ED, transfer or planned), intensive care unit stay, and patient-directed discharge. Patient-directed discharge was defined as hospital separations coded as left against medical advice and ED presentations coded as left at own risk, either without treatment, after clinical advice regarding treatment options, or after treatment started. National Death Index data were used to collect all-cause mortality within 100 days of hospital separation.

### Statistical methods

Descriptive analyses summarized cohort characteristics and the period prevalence of ED presentations and hospital separations with an IRI. Period prevalence was estimated for any IRI and each IRI for both ED presentations and hospital separations, calculated by dividing the number of participants who had at least one ED presentation or hospital separation with an IRI by the total number of participants from 2008 to 2018. Individuals who had multiple IRIs were captured across each IRI prevalence estimate, regardless of whether the IRI occurred in the same hospital separation episode.

The start of follow-up was the participant’s self-report initiation of injecting drug use or 1 January 2008, whichever came last. In the cohort study’s annual follow-up surveys, participants reported dates of injecting drug use cessation and resumption, if applicable. Therefore, the end of follow-up was the participant’s date of death, self-report cessation of injecting drug use without resumption reported in a subsequent follow-up survey, or 31 December 2018, whichever came first. Overall and annual incidence were calculated by dividing the number of ED presentations and hospital separations for each IRI and any IRI by the number of person-years in the cohort during the specified period (expressed as IRI events per 1,000 person-years). Individuals who had multiple IRIs within one hospital separation were captured across each IRI incidence estimate.

We also excluded individuals’ person-time follow-up period where injecting drug use ceased for 12 months or longer; therefore, ED presentations and hospital separations with an IRI during this period were excluded. As our analysis was a count of event over person-time, individuals were not censored after an IRI event unless they died or ceased injecting for 12 months or longer. Due to privacy requirements of the data custodian, we were unable to report central nervous system and deep abscess IRIs, annual ED presentations stratified by SSTI and invasive infection, or inpatient mortality with an IRI. All the statistical analyses were performed by using R version 4.05 [22].

## Results

### Participants

**Figure 1.**
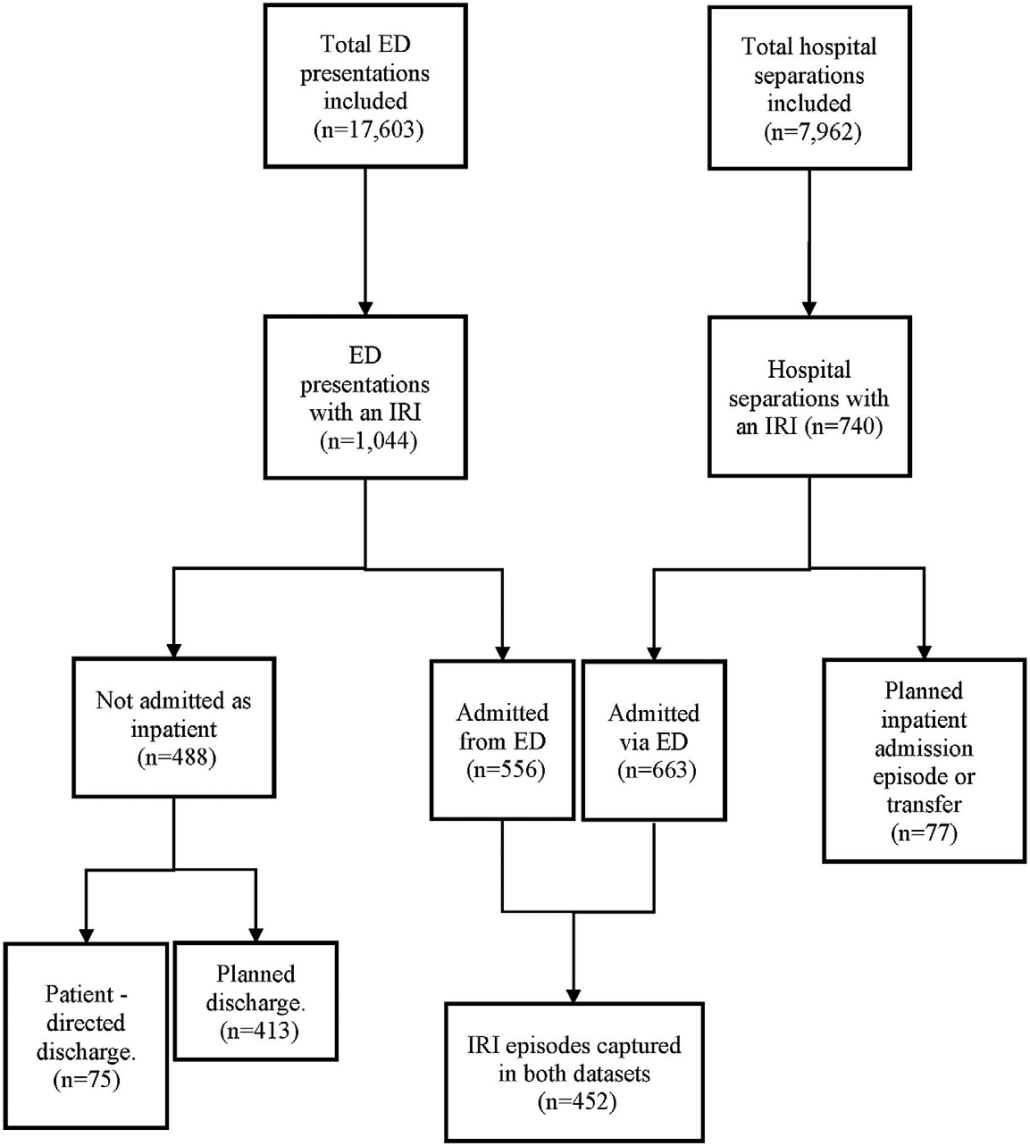
Flowchart of emergency department presentations and hospital separations with an injecting-related infection.

Among 1,288 participants, 427 people (33%) presented to ED with ≥1 IRI (1,044 ED presentations in total) and 345 (27%) were admitted to hospital with ≥1 IRI (740 hospital separations in total). Most hospital separations (n = 663, 89.6%) with an IRI were reported as admitted via an ED presentation, of which only 68.2% (n = 452) were captured in the ED presentation dataset (Figure 1). Both ED presentations and hospital separations with an IRI were more commonly male, 67% and 64%, respectively, and were similar in age (median age of 32 years (interquartile range (IQR): 28–36) and 32 years (IQR: 29–36), respectively). Patient-directed discharge for patients with an IRI occurred for 7.2% (n = 75) of ED presentations and 20.5% (n = 152) of hospital separations. Characteristics of ED presentations and hospital separations with an IRI are presented in Table 1.

**Table 1.** Characteristics of participant hospitalizations with an injecting-related infection, by emergency department presentations and hospital separations

Participant characteristics	ED presentations with ≥ 1 IRI (*n* = 1,044)	Hospital separations with ≥ 1 IRI (*n* = 740)
Unique patients	427 (33.2)	345 (26.8)
Age at hospitalization, median [IQR]	32 [28, 36]	32 [29, 36]
Age at hospitalization		
≤25	130 (12.5)	63 (8.5)
26–30	303 (29.0)	216 (29.2)
31–35	339 (32.4)	254 (34.3)
36–45	216 (20.7)	166 (22.4)
>45	56 (5.4)	41 (5.5)
Male sex	696 (66.7)	472 (63.8)
Born in Australia	856 (82.0)	599 (80.9)
Aboriginal and Torres Strait Islander	158 (15.1)	94 (12.7)
ED presentation in previous 30 days	464 (44.4)	411 (55.5)
Hospital separation in the past 30 days	189 (18.1)	227 (30.7)
Intensive care unit stay	–	65 (0.8)
Length of stay (days), median [IQR]	–	3 [1, 8]
Patient-directed discharge	75 (7.2)	152 (20.5)
All-cause mortality within 100 days of hospitalization	8 (0.8)	11 (1.5)

Abbreviations: ED, emergency department; IRI, injecting-related infection; IQR, interquartile range.

### Frequency, period prevalence and incidence of ED presentation and hospital separations with IRIs

The period prevalence of any ED presentation and hospital separation involving an IRI over the observation period was 33% (95%CI: 31%–36%) and 27% (95%CI: 24%–29%), respectively (Table 2). Most ED presentations and hospital separations involved a SSTI, 89% (n = 919) and 75% (n = 552), respectively. Of 128 ED presentations involving invasive infection, most were for bloodstream infection/sepsis (n = 80, 63%), followed by osteomyelitis/septic arthritis (n = 30, 23%) and infective endocarditis (n = 18, 16%). Of 250 hospital separations involving invasive infection, most were for bloodstream infection/sepsis (n = 157, 63%), followed by osteomyelitis/septic arthritis (n = 92, 37%) and infective endocarditis (n = 80, 32%). More than one IRI was coded for 126 (18%) hospital separations.

**Table 2.** Frequency, period prevalence and incidence of emergency department presentations and hospital separations with an injecting-related infection

Type of IRI	Emergency department presentations				Hospital separations					
	Total presentations, *N*	Unique participants, *N*	Period prevalence, % [95% CI]	Incidence, per 1,000 person-years [95% CI]	Total separations, *N*	Unique participants, *N*	Period prevalence, % [95% CI]	Incidence, per 1,000 person-years [95% CI]	Bed days, *N*	Length of stay, median [IQR]
Any IRI	1,044	427	33.2 [30.6–35.8]	80 [75–85]	740	345	26.8 [24.4–29.3]	56 [52–61]	7,715	3 [1–8]
Skin or soft tissue infection Uncomplicated Complicated	919 – –	387 – –	30.1 [27.6–32.7] – –	70 [66–75] – –	552 490 62	282 261 48	21.9 [19.7–24.3] 20.3 [18.1–22.6] 3.7 [2.8–5.0]	42 [39–46] 37 [34–41] 5 [4–6]	4,670 3,527 1,143	2 [1–5] 2 [1–4] 10 [3–28]
Invasive infection Bloodstream infection/sepsis Osteomyelitis/septic arthritis Infective endocarditis	128 80 30 18	94 60 23 17	7.3 [5.9–8.9] 4.7 [3.6–5.9] 1.8 [1.2–2.7] 1.3 [0.8–2.2]	10 [8–12] 6 [5–8] 2 [1–3] 1 [0.8–2]	250 157 92 80	141 105 48 51	10.9 [9.3–12.8] 8.2 [6.7–9.8] 3.7 [2.8–5] 4.0 [3.0–5.2]	19 [17–22] 12 [10–14] 7 [6–9] 6 [5–8]	3,632 2,470 1,516 1,311	8 [2–18] 10 [3–24] 9 [3–25] 10 [3–26]

Abbreviations: CI, confidence interval; ED, emergency department; IRI, injecting-related infection; IQR, interquartile range.

From 2008 to 2018, across 13,106 person-years of follow-up (median 11 years (range 0.8–11 and IQR: 11–11); mean 10.2 years (standard deviation: 2.04) per participant), the incidence of ED presentations with an IRI was 80 per 1,000 person-years, and 56 per 1,000 person-years for hospital separations. The incidence of ED presentations involving an SSTI was 70 per 1,000 person-years and involving invasive infection was 10 per 1,000 person-years. The incidence of hospital separations involving SSTI was 42 per 1,000 person-years and involving invasive infection was 19 per 1,000 person-years. There were a total 7,159 bed-days with an IRI, and the median inpatient length of stay was 3 days (range 0–980 and IQR: 1–8).

### Annual incidence of ED presentation and hospital separations with an IRI

**Figure 2.**
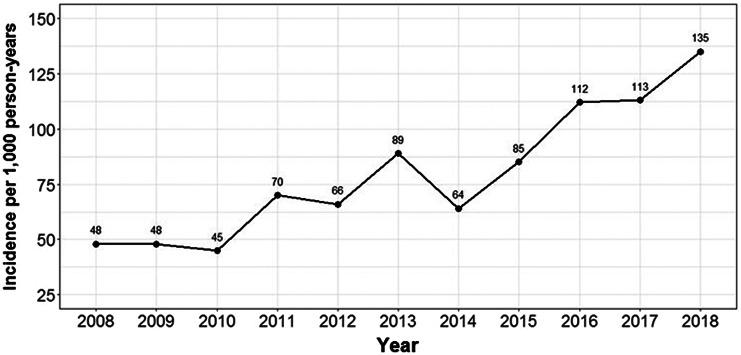
Incidence of emergency department presentations with an injecting-related infection per 1,000 person-years.

**Figure 3.**
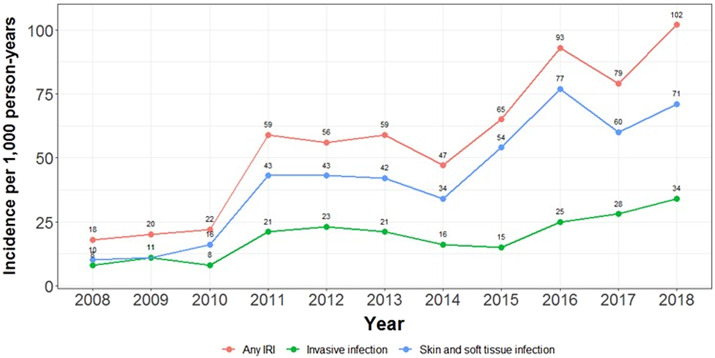
Incidence of hospital separations with an injecting-related infection per 1,000 person-years.

There was a steady increase in the incidence of ED presentations and hospital separations with an IRI over time (Figures 2 and 3). From 2008 to 2018, incidence of both ED presentations and hospital separations with an IRI increased 2.8 times from 48 to 135 per 1,000 person-years and 5.7 times from 18 to 102 per 1,000 person-years, respectively. Over this period, the increase in hospital separations was observed for both SSTIs, 7.1 times from 10 to 71 per 1,000 person-years, and invasive infections, 4.3 times from 8 to 34 per 1,000 person-years. Supplementary Figures S1 and S2 present the annual number of unique participants with an IRI-related ED presentation and hospital separation, illustrating an increasing number of unique participants presenting to hospital with an IRI annually since 2008.

## Discussion

We observed a high 11-year period prevalence and incidence of ED presentations and hospital separations with an IRI among people who inject drugs in Australia, with both ED presentations and hospital separations increasing in recent years. Over the study period, one-third of our cohort presented to an ED and over a quarter were hospitalized with an IRI, most commonly for SSTI. From 2008 to 2018, the incidence of ED presentations and hospital separations with an IRI increased substantially and patient-directed discharge was also common.

We report a high period prevalence and increasing incidence of both unique individuals and overall count of ED presentations and hospital separations with SSTI. These infections can often be managed in primary care, and hospitalization may represent delayed access to care within this cohort [23–25]. In Victoria, Australia, there are multiple points for intervention to prevent and treat SSTI before hospital presentation and admission, including population-specific primary care centres, that provide a range of important services including SSTI treatment [26]. These services are an important engagement point for this population who traditionally underutilize primary care services. Nevertheless, despite the existence of these services, we found an increase in SSTIs over time. We suspect that the impact of these services could be improved by increasing staffing and operating hours of existing services, and the establishment of new services. Furthermore, there is also a clear need for upstream prevention and early treatment of IRIs, particularly SSTIs, that could potentially reduce the high incidence of ED presentations and hospital separations observed in our study [27]. Prevention efforts are also required to address causes of IRIs, such as education to improve hygiene and injecting techniques, and addressing social-structural determinants that limit access to, and storage of, sterile injecting equipment and environmental hygiene, such as homelessness and poverty [28, 29].

The prevalence of hospital separations with an IRI observed in this cohort over 11 years (2008 to 2018) was similar to that in a cohort of people who injected heroin entering community-based substance use treatment in South London over 12 years (2006 to 2017); however, the overall incidence of hospital separations with an IRI was higher than observed in our study [11]. We report both a higher period prevalence and incidence of hospital separations with an IRI than observed in a cohort of people with a history of opioid agonist treatment in New South Wales over 17 years (2001 to 2017) [13]. Similar to a retrospective analysis of hospitalizations among patients with a diagnosis code for substance use and a serious bacterial infection in Oregon, we report that the number of unique individuals with an IRI has also been increasing in recent years [7]. In Victoria, Australia, there is consistently high needle syringe coverage and there has not been changes in injecting frequency in our cohort; therefore, the increase in unique individuals with an IRI may be due to other factors such as an ageing cohort and changes in social-structural determinants [30]. Our period prevalence estimates of invasive infections were higher than observed in South London and New South Wales [11, 12], which may be driven by higher engagement in health services leading to early treatment of IRIs by the cohorts engaged in drug treatment, compared to our community-recruited cohort [17]. Overall, the high period prevalence of invasive IRIs reiterates that people who inject drugs are at high risk for life-threatening severe infections and that cohorts recruited from the community are at even higher risk than samples recruited from drug treatment [12, 17].

Hospitals have been identified as a unique risk environment for people who inject drugs as they can experience stigma, inadequate pain, and withdrawal management and isolation from social supports when admitted to hospital [31, 32]. This may lead to patient-directed discharge, which presents a lost opportunity for care, may lead to poorer patient outcomes, including readmission and increased mortality, and increase costs to the public health system [33]. In our study, 21% of people were observed to patient-directed discharge which is consistent with previous estimates on patient-directed discharge in people hospitalized with an IRI (range 13–30%) [11, 12, 34]. Hospital care is nearly always required for invasive infections; however, the implementation of flexible patient-centred models which focus on hospital-based harm reduction for people who inject drugs may mitigate the consequences of patient-directed discharge, such as supervised injecting services, use of nicotine replacement therapy, short-acting opioids to treat withdrawal, and opioid agonist treatment initiation [35, 36]. In addition, these hospital-based interventions can be supported by community-based clinical care such as safe discharge with oral antibiotic therapy or weekly infusions and outpatient programmes which have emerging evidence of success [37–40].

Our findings highlight the importance of early intervention in primary care to prevent hospitalization for SSTIs and for hospital-based harm reduction interventions and safe discharge into community-based clinical care to mitigate patient-directed discharge. Further efforts are required to bridge the gap between clinical care and the needs of people who inject drugs, and all interventions should be supported by strategies which are known to be effective at reducing drug-related harms including SSTI, such as needle and syringe coverage. Further investigation is required to understand the causes of increasing IRIs, risk factors for hospitalization with an IRI, and the sequalae of patients that patient-directed discharge to better understand the implications and guide future clinical practice and policy.

### Limitations

This study has some limitations. First, not all acute infections may be attributable to injecting practices; however, for people who inject drugs, SSTI are consistently reported as one of the most common causes of ED presentations and hospital admissions, and invasive infections occur more frequently in this cohort compared to the general population [3, 14, 20]. Second, one infection episode could be captured multiple times for those that patient-directed discharged and represented or were readmitted, and for patient with multiple IRIs coded within one hospital separation (i.e., infective endocarditis and bloodstream infection/sepsis); however, we aimed to estimate incidence of ED presentations and hospital separations with any and each IRI rather than total unique IRI episodes. Third, due to privacy requirements, aggregate counts below a threshold were unable to be reported, including central nervous system and deep abscess IRIs, annual ED presentations stratified by SSTI and invasive infection, or inpatient mortality with an IRI. Fourth, data after 2019 were not available; therefore, ongoing analysis should be explored to confirm if IRIs have continued to increase in recent years. Fifth, our study is restricted to ED presentations and hospital separations in Victoria, Australia, and does not include hospital data from other jurisdictions in Australia. Finally, there may be limitations on the generalizability of our findings outside of Victoria or internationally, as local injecting drug use and health service utilization may be specific to this area, and Australia’s universal healthcare system includes subsidized and/or free hospital care and people who inject drugs may present to hospital more regularly than in settings where hospital care is not available for free. However, people who inject drugs are often reluctant to access medical care, and a lack of universal health care in other settings may further deter this population from receiving care for life-threatening infections, as demonstrated in our cohort’s high incidence which suggests that universal health care is necessary but not sufficient.

## Conclusion

We have provided novel and objective epidemiological data of ED presentation and hospital separations with an IRI in a cohort of people who inject drugs in Melbourne, Australia. Our findings are consistent with current evidence that IRI hospitalization have been increasing in recent years, and we observed the highest incidence of ED presentations and hospital separations with an IRI reported in Australia. Combined with high prevalence of patient-directed discharge in this population, findings indicate a need for better understanding of the factors driving this increase, and integrated interventions to address the burden of IRIs.

## Supporting information

Curtis et al. supplementary material Curtis et al. supplementary material

## Data Availability

All data used in this study are protected under the privacy policies of Victorian Department of Health and Human Services ‘Deed of Acknowledgment and Confidentiality’. Signed confidentiality agreements prevent us from sharing the data.

## Supplementary material

For supplementary material accompanying this paper visit http://doi.org/10.1017/S0950268823001784. click here to view supplementary material

## References

[1] Hope V (2010) Neglected infections, real harms: a global scoping of injection-related bacterial infections and responses. The Global State of Harm Reduction 89–95.

[2] Larney S, et al. (2017) A systematic review of injecting-related injury and disease among people who inject drugs. Drug and Alcohol Dependence 171, 39–49. 10.1016/j.drugalcdep.2016.11.029 28013096

[3] Hope V, et al. Healthcare seeking and hospital admissions by people who inject drugs in response to symptoms of injection site infections or injuries in three urban areas of England. Epidemiology and Infection 143, 120–131. 10.1017/S0950268814000284 PMC9206814 24568684

[4] Magsino K, et al. (2018) Treatment outcomes for right-sided endocarditis in intravenous drug users: a systematic review and analysis of outcomes in a tertiary centre. The Journal of Thoracic and Cardiovascular Surgery 55, 552–562. 10.1055/s-0037-1618578 29351694

[5] Valerio H, et al. (2021) Opportunities to enhance linkage to hepatitis C care among hospitalized people with recent drug dependence in New South Wales, Australia: a population-based linkage study. Clinical Infectious Diseases 73, 2037–2044. 10.1093/cid/ciab526 34107022

[6] Ciccarone D, et al. (2016) Nationwide increase in hospitalizations for heroin-related soft tissue infections: associations with structural market conditions. Drug and Alcohol Dependence 163, 126–133. 10.1016/j.drugalcdep.2016.04.009 27155756 PMC4881875

[7] Capizzi J, et al. (2020) Population-based trends in hospitalizations due to injection drug use-related serious bacterial infections, Oregon, 2008 to 2018. PLoS One 15, 1–17. 10.1371/journal.pone.0242165 PMC7652306 33166363

[8] McCarthy NL, et al. (2020) Bacterial infections associated with substance use disorders, large cohort of United States hospitals, 2012–2017. Clinical Infectious Diseases 71, 37–44. 10.1093/cid/ciaa008 PMC7900878 31907515

[9] Wurcel AG, et al. (2020) Increasing infectious endocarditis admissions among young people who inject drugs. Open Forum Infectious Diseases 3, 1–5. 10.1093/ofid/ofw157 PMC5084714 27800528

[10] Low ZM, et al. (2020) Burden of infective endocarditis in an Australian cohort of people who inject drugs. Internal Medicine Journal 50, 1240–1246. 10.1111/imj.14717 31841254

[11] Lewer D, et al. (2020) Incidence and treatment costs of severe bacterial infections among people who inject heroin: a cohort study in South London, England. Drug and Alcohol Dependence 212, 108057. 10.1016/j.drugalcdep.2020.108057 32422537 PMC7301433

[12] Colledge-Frisby S, et al. (2022) The impact of opioid agonist treatment on hospitalisations for injecting-related diseases among an opioid dependent population: a retrospective data linkage study. Drug and Alcohol Dependence 236, 109494. 10.1016/j.drugalcdep.2022.109494 35605532

[13] Curtis SJ, et al. (2022) Hospitalisation with injection‐related infections: validation of diagnostic codes to monitor admission trends at a tertiary care hospital in Melbourne, Australia. Drug and Alcohol Review 51, 1053–1061. 10.1111/dar.13471 35411617

[14] Wilson J, et al. (2022) Victorian drug trends 2021: key findings from the Illicit Drug Reporting System (IDRS) Interviews. Sydney, NSW, Australia: National Drug and Alcohol Research Centre, UNSW Sydney.

[15] Dahlman D, et al. (2018) Both localized and systemic bacterial infections are predicted by injection drug use: a prospective follow-up study in Swedish criminal justice clients. PLoS One 13, 0196944. 10.1371/journal.pone.0196944 PMC5979029 29851980

[16] Heard S, Iversen J, Maher L (2022) Australian Needle Syringe Program Survey National Data Report 2017–2021: Prevalence of HIV, HCV and Injecting and Sexual Behaviour among NSP Attendees. Sydney, NSW, Australia: Kirby Institute, UNSW Sydney.

[17] Mittal ML, et al. (2019) Opioid agonist treatment and the process of injection drug use initiation. Drug and Alcohol Dependence 197, 354–360. 10.1016/j.drugalcdep.2018.12.018 30922483 PMC6719710

[18] Van Den Boom W, et al. (2022) Cohort profile: the Melbourne injecting drug user cohort study (SuperMIX). International Journal of Epidemiology 51, 123–130. 10.1093/ije/dyab231 34961882

[19] Horyniak D, et al. (2013) Establishing the Melbourne injecting drug user cohort study (MIX): rationale, methods, and baseline and twelve-month follow-up results. Harm Reduction Journal 10, 1–4. 10.1186/1477-7517-10-11 23786848 PMC3691755

[20] Nambiar D, et al. (2017) A prospective cohort study of hospital separations among people who inject drugs in Australia: 2008–2013. BMJ Open 7, 014854. 10.1136/bmjopen-2016-014854. PMC5724200 28821513

[21] Di Rico R, et al. (2018) Drug overdose in the ED: a record linkage study examining emergency department ICD-10 coding practices in a cohort of people who inject drugs. BMC Health Services Research 188, 1–9. 10.1186/s12913-018-3756-8 PMC6282274 30518362

[22] RStudio: Integrated Development for R, version 4.0.2. Available at http://www.rstudio.com (accessed 4 October 2022).

[23] Islam MM, et al. (2013) Healthcare utilisation and disclosure of injecting drug use among clients of Australia’s needle and syringe programs. Australian and New Zealand Journal of Public Health 37, 148–154. 10.1111/1753-6405.12032 23551473

[24] Nambiar D, Stoové M, Dietze P (2014) A cross-sectional study describing factors associated with utilisation of GP services by a cohort of people who inject drugs. BMC Health Services Research 14, 1–7. 10.1186/1472-6963-14-308 25030526 PMC4110070

[25] Neale J, Tompkins C, Sheard L (2007) Barriers to accessing generic health and social care services: a qualitative study of injecting drug users. Health & Social Care in the Community 16, 147–154. 10.1111/j.1365-2524.2007.00739.x 18290980

[26] Department of Health. Available at https://www.health.vic.gov.au/aod-treatment-services/needle-and-syringe-program (accessed 4 October 2022).

[27] Sanchez DP, et al. (2021) Wounds and skin and soft tissue infections in people who inject drugs and the utility of syringe service programs in their management. Advances in Wound Care 10, 571–582. 10.1089/wound.2020.1243 33913781 PMC8312019

[28] Ivan M, et al. (2016) Reducing injecting-related injury and diseases in people who inject drugs: results from a clinician-led brief intervention. Australian Family Physician 45, 129–133. 27052050

[29] Harris M, et al. (2020) Navigating environmental constraints to injection preparation: the use of saliva and other alternatives to sterile water among unstably housed PWID in London. Harm Reduction Journal 17, 1–11. 10.1186/s12954-020-00369-0 32276626 PMC7145770

[30] Colledge-Frisby S, et al. (2023) Injection drug use frequency before and after take-home naloxone training. JAMA Network Open 6, 2327319–2327319. 10.1001/jamanetworkopen.2023.27319 PMC10403778 37540514

[31] Pollini R, et al. (2021) A qualitative assessment of discharge against medical advice among patients hospitalized for injection-related bacterial infections in West Virginia. International Journal of Drug Policy 94, 103206. 10.1016/j.drugpo.2021.103206 33765516 PMC8373672

[32] McNeil R, et al. (2014) Hospitals as a ‘risk environment’: an ethno-epidemiological study of voluntary and involuntary discharge from hospital against medical advice among people who inject drugs. Social Science & Medicine 105, 59–66. 10.1016/j.socscimed.2014.01.010 24508718 PMC3951660

[33] Southern WN, Nahvi S, Arnsten JH (2012) Increased risk of mortality and readmission among patients discharged against medical advice. The American Journal of Medicine 125, 594–602. 10.1016/j.amjmed.2011.12.017 22513194 PMC3372411

[34] Langham FJ, et al. (2022) Acute injection‐related infections requiring hospitalisation among people who inject drugs: clinical features, microbiology and management. Drug and Alcohol Review 41, 1543–1553. 10.1111/dar.13525 36053863 PMC9804300

[35] Kleinman RA, Wakeman SE (2021) Treating opioid withdrawal in the hospital: a role for short-acting opioids. American College of Physicians 175, 283–284. 10.7326/M21-3968 34807718

[36] Buresh M, et al. (2022) Adapting methadone inductions to the fentanyl era. Journal of Substance Abuse Treatment 141, 108832. 10.1016/j.jsat.2022.108832 35870437

[37] Marks LR, et al. (2020) Evaluation of partial oral antibiotic treatment for persons who inject drugs and are hospitalized with invasive infections. Clinical Infectious Diseases 71, 650–656. 10.1093/cid/ciaa365 PMC7745005 32239136

[38] Suzuki J, et al. (2018) Outpatient parenteral antimicrobial therapy among people who inject drugs: a review of the literature. Open Forum Infectious Diseases 5, 1–9. 10.1093/ofid/ofy194 PMC6127783 30211247

[39] Appa A, Barocas JA (2022) Can I safely discharge a patient with a substance use disorder home with a peripherally inserted central catheter? NEJM Evidence 1, 1–5. 10.1056/EVIDccon2100012 38319183

[40] Baddour LM, et al. (2022) Management of infective endocarditis in people who inject drugs: a scientific statement from the American Heart Association. Circulation 146, 187–201. 10.1161/CIR.0000000000001090 36043414

