# Supplementary material for: Prevalence and incidence of emergency department presentations and hospital separations with injecting-related infections in a longitudinal cohort of people who inject drugs
Source: Epidemiol Infect. 2023 Nov 13;151:e192. doi: 10.1017/S0950268823001784 (PMC10728979; doi:10.1017/S0950268823001784)

Epidemiology and Infection, Prevalence and incidence of emergency department presentations and hospital separations with injecting-related infections in a longitudinal cohort of people who inject drugs, Curtis, S.J., Colledge-Frisby S., Stewardson, A.J., Doyle, J.S., Higgs, P., Hickman, M., Maher, L., Stoové, M.A., Dietze, P.M.

**Supplementary Material**

**Table S1. International Classification of Diseases, Tenth Revision, Australian Modification (ICD-10-AM) codes used to identify emergency department presentations and hospital separations with injecting-related infections.**

| **IRI** | **ICD-10-AM codes** |
| --- | --- |
| **Skin or soft tissue infections** | A46 Erysipelas  A48.0* Gas gangrene  B43.2 Subcutaneous phaeomycotic abscess and cyst  L01* Impetigo  L02* Cutaneous abscess, furuncle, and carbuncle  L03* Cellulitis  L04* Acute lymphadenitis  L08* Other local infections of skin and subcutaneous tissue  M65.0 Abscess of tendon sheath  M72.6 Necrotizing fasciitis |
| **Bloodstream infections or sepsis** | A27 Actinomycotic sepsis  A40* Streptococcal sepsis  A41* Other sepsis  A49* Bacterial infection of unspecified site  B37.7 Candidal sepsis  I26.01 Septic pulmonary embolism with acute cor pulmonale  I26.90 Septic pulmonary embolism without acute cor pulmonale  R57.2 Septic shock  R65.1 Severe sepsis |
| **Infective endocarditis** | B37.6 Candidal endocarditis  I33* Acute and subacute endocarditis  I34* Nonrheumatic mitral valve disorders  I35* Nonrheumatic aortic valve disorders  I36* Nonrheumatic tricuspid valve disorders  I37* Pulmonary valve disorders  I38 Endocarditis, valve unspecified  I39* Endocarditis and heart valve disorders in diseases classified elsewhere |
| **Osteomyelitis or septic arthritis** | G06.1 Intraspinal abscess and granuloma  M00* Pyogenic arthritis  M86* Osteomyelitis  M46.2 Osteomyelitis of vertebra  M46.3 Infection of intervertebral disc (pyogenic)  M46.5 Other infective spondylopathies |
| **Includes all ICD-10-AM codes starting with.* | |

**Figure S1. Annual count of unique participants with an emergency department presentation for an injecting-related infection in the SuperMIX cohort**
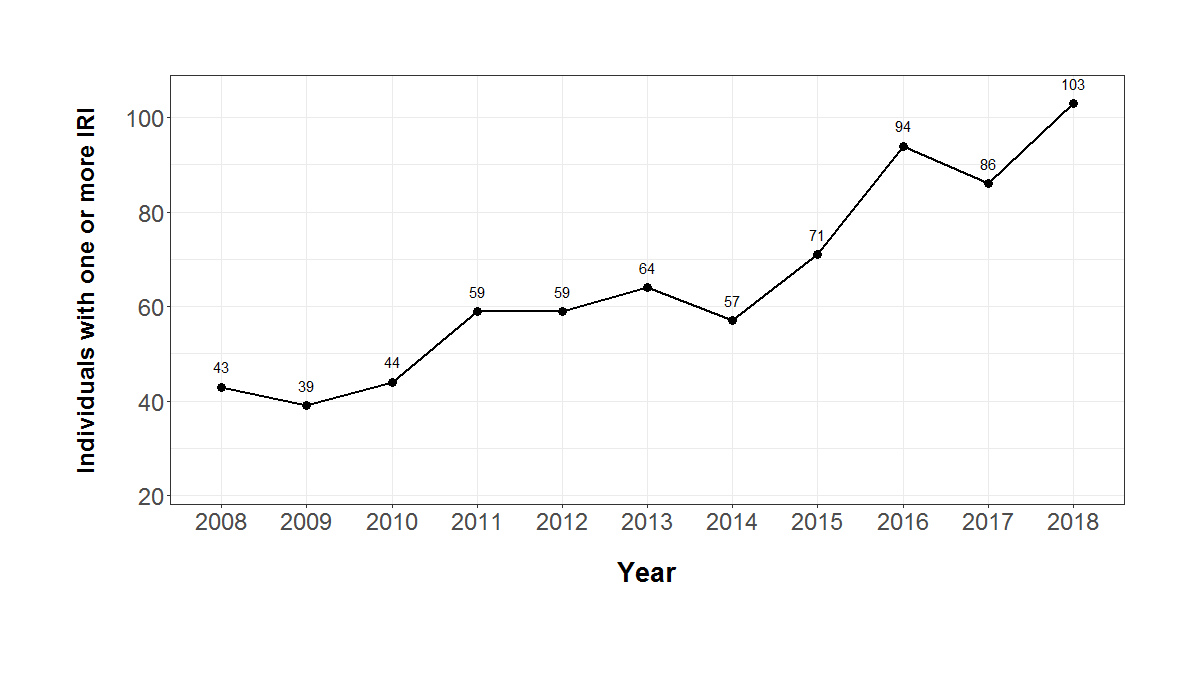


**Figure S2. Annual count of unique participants with a hospital separation for an injecting-related infection in the SuperMIX cohort**
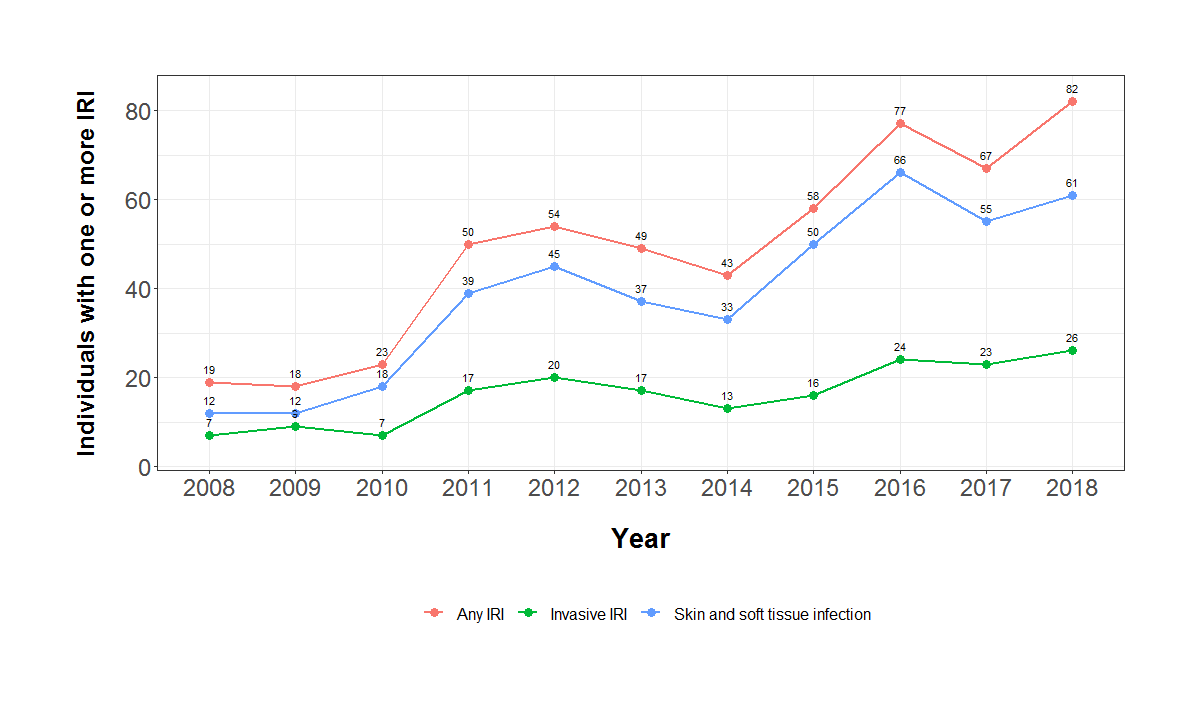

Supplement: Curtis et al. supplementary material [file S0950268823001784sup001.docx]
